# Comprehensive Analysis of METTLs (METTL1/13/18/21A/23/25/2A/2B/5/6/9) and Associated mRNA Risk Signature in Hepatocellular Carcinoma

**DOI:** 10.1155/2023/6007431

**Published:** 2023-10-12

**Authors:** Haoyu Wang, Shangshang Hu, Junjie Nie, Xiaodan Qin, Xu Zhang, Qian Wang, John Zhong Li

**Affiliations:** ^1^The Key Laboratory of Rare Metabolic Disease, Department of Biochemistry and Molecular Biology, The Key Laboratory of Human Functional Genomics of Jiangsu Province, Key Laboratory of Targeted Intervention of Cardiovascular Disease, Collaborative Innovation Center for Cardiovascular Disease Translational Medicine, Nanjing Medical University, Nanjing 211166, China; ^2^Department of Clinical Laboratory Diagnostics, School of Medicine, Southeast University, Nanjing 210009, China; ^3^Department of Laboratory Medicine, Nanjing First Hospital, Nanjing Medical University, Nanjing 210006, China

## Abstract

Currently, 80%–90% of liver cancers are hepatocellular carcinomas (HCC). HCC patients develop insidiously and have an inferior prognosis. The methyltransferase-like (METTL) family principal members are strongly associated with epigenetic and tumor progression. The present study mainly analyzed the value of METTLs (METTL1/13/18/21A/23/25/2A/2B/5/6/9) and associated mRNA risk signature for HCC. METTLs expression is upregulated in HCC and is a poor prognostic factor in HCC. METTLs were upregulated in patients older than 60 and associated with grade. Except for METTL25, the remaining 10 genes were associated with the HCC stage, invasion depth (*T*). In addition, METTLs showed an overall alteration rate of 50%. Except for METTL13/2A/25/9, the expression of the other seven genes was significantly associated with overall survival, disease-specific survival, and progression-free survival. Multivariate studies have shown that METTL21A/6 can be an independent prognostic marker in HCC. A total of 664 mRNAs were selected based on Pearson correlation coefficient (*R* > 0.5), unsupervised consensus clustering, weighted coexpression network analysis, and univariate Cox analysis. These mRNAs were significantly associated with METTLs and were poor prognostic factors in HCC patients. The least absolute shrinkage and selection operator (lasso) was used to construct the best METTLs associated with mRNA risk signature. The mRNA risk signature was significantly associated with age, stage, and *t* grade. The mRNA high-risk group had higher TP53 and RB1 mutations. This study constructed a nomogram with the mRNA risk profile and clinicopathological features, which could better predict the OS of individuals with HCC. We also analyzed associations between METTLs and mRNA risk signatures in epithelial-mesenchymal transition, immune checkpoints, immune cell infiltration, tumor mutational burden, microsatellite instability, cancer stem cells, tumor pathways, and drug sensitivity. In addition, this study constructed a protein interaction network network including METTLs and mRNA risk signature genes related to tumor microenvironment remodeling based on single-cell sequencing. In conclusion, this study provides a theoretical basis for the mechanism, biomarker screening, and treatment of HCC.

## 1. Introduction

Hepatocellular carcinoma (HCC) is one of the digestive tract tumors and an aggressive type of tumor [1]. Although HCC incidence ranks sixth worldwide, HCC mortality ranks third worldwide [2]. Currently, the diagnosis of HCC is limited, resulting in the fact that most patients with HCC are advanced stage when diagnosed [3]. In addition, HCC treatment approaches are also limited, and patients with advanced HCC who are treated also develop tumor cell metastasis, ultimately leading to patient death [4]. Therefore, finding effective biomarkers and therapeutic targets for HCC is crucial.

Aberrant expression of a gene may promote or suppress HCC progression depending on whether this gene is an oncogene or a tumor suppressor [5]. Human methyltransferase-like (METTL) proteins are a superfamily of S-adenosylmethionine—dependent enzymes. METTL's primary function is to transfer methyl groups onto various molecules, including lipids, proteins, and nucleic acids [6]. Studies have shown that multiple members of METTL are strongly associated with tumor progression [7]. METTL1 is one of the significant regulators of N7-methylguanosine (m7G), which can form a complex with wdr4 to mediate m7G tRNA modification and, in turn, promote the progression of multiple tumors, including lung cancer [8], bladder cancer [9], and liver cancer [10]. METTL3/4/5/13/14/16 are N6-methyladenosine (m6A) regulators, and they promote tumor progression through m6A methylation modification [11]. Moreover, METTL6/7b/9/11A/21B/18 [12–17] was able to act as an oncogene to promote tumor progression. However, METTLs have yet to be systematically studied in HCC.

Among the 33 METTL family members, 11 METTLs (METTL1/13/18/21A/23/25/2A/2B/5/6/9) with upregulated expression in HCC and poor prognosis for HCC patients were enrolled in this study. In addition, this study employed multiple algorithms to construct the mRNA risk signature associated with these 11 genes separately. Ultimately, this study comprehensively analyzed the correlation between METTLs and mRNA risk signatures in terms of prognosis, clinicopathological features, mutations, tumor microenvironment (TME), epithelial-mesenchymal transition (EMT), immune cell infiltration, tumor mutational burden (TMB), microsatellite instability (MSI), cancer stem cells (RNAss), cancer pathways, and drug sensitivity. In conclusion, this study provides a theoretical basis for the prognosis, biomarker screening, mechanism, and drug screening of HCC.

## 2. Materials and Methods

### 2.1. Expression Data and Clinical Data Acquisition

HCC expression data were obtained from The Cancer Genome Atlas (TCGA) (tumor: *n* = 374, normal: *n* = 50), Genotype-Tissue Expression (GTEx) (normal: *n* = 110), and Gene Expression Omnibus (GEO) databases. The GEO databases included GSE54236 (tumor: *n* = 81, normal: *n* = 80) and GSE64041 (tumor: *n* = 60, normal: *n* = 60). This study combined TCGA and GTEX data into new TCGA_ GTEx datasets (tumor: *n* = 374, normal: *n* = 160). The R software “limma” and “ggplot2” packages were used for differential analysis and visualization. Receiver operating characteristic (ROC) curves were plotted using the R software “pROC” package. Clinical data of HCC were obtained from the TCGA database and International Cancer Genome Consortium (ICGC) database, including survival time (*n* = 370), age (*n* = 376), gender (*n* = 377), grade (*n* = 376), tumor stage (*n* = 354), invasion depth (*T*) (*n* = 374), lymph node metastasis (*n*) (*n* = 376), and distant metastasis (*M*) (*n* = 261). The differences between gene expression or risk scores and clinicopathological characteristics were analyzed using R software “reshape2” and “ggplot2” packages.

### 2.2. Mutational Analysis and Prognostic Analysis of METTLs

METTLs genes were accessed using the Cbioportal site (http://www.cbioportal.org/) [18]. Prognostic analysis of METTLs in HCC patients was performed using R software “survival,” “survminer,” “regplotz,” and “RMS” packages, including overall survival (OS), disease-specific survival (DSS), progression-free interval (PFI), and univariate/multivariate Cox regression analysis. Multivariate Cox regression analysis parameters included METTLs, age, grade score, *T*, and stage.

### 2.3. Acquisition of mRNAs Closely Associated with METTLs

First, Pearson correlation analysis in the R software “limma” package was used to screen the related mRNAs of METTLs in this study (Pearson *r* > 0.5 and *P* < 0.05). Unsupervised consensus clustering is an algorithm for *k*-means machine learning [19]. The present study subsequently employed unsupervised consensus clustering to analyze the TCGA cohort based on the expression of METTLs. The R software “ConsensusClusterPlus” package was used for the clustering. And OS curves were used to evaluate the prognosis of different clusters for HCC patients. The R software “limma” and “ggplot2” packages were adopted to screen the differential mRNAs between different clusters (|log FC| > 0.585, *P* < 0.05). This study further used R software “limma” and the “WGCNA” package to construct a coexpression network of 11 genes in the METTL family based on weighted gene coexpression network analysis (WGCNA) [20]. The present study intersected the relevant mRNAs of 11 genes in the METTL family, the upregulated mRNAs in the subcohort, and the coexpressed mRNAs in the WGCNA. The intersection genes were further screened in this study by univariate Cox analysis. This study defines mRNAs with poor prognosis for HCC as genes closely related to METTLs. Finally, “clusterProfiler” and “org. HS. Eg.db” packages were adopted to perform Kyoto Encyclopedia of Genes and Genomes (KEGG) and GO enrichment analysis of closely related mRNAs of METTLs.

### 2.4. Construction of an mRNA Risk Signature

The present study constructed an associated risk signature based on the closely related mRNAs of METTLs. This study employed R software “glmnet” and “survival” packages for lasso regression and multivariate Cox analysis to construct the most suitable risk signature. The risk score for patients was calculated from the normalized expression level of each gene and the corresponding regression coefficient with the formula: Risk score = ∑coefi  ^*∗*^ exp. The R software “survival,” “survminer,” and “survminer” packages were used to draw the OS curves and time-dependent ROC curves for the mRNA risk signature. The R software “Rtsne” and “ggplot2” packages were used to draw principal component analysis (PCA) and t-SNE scatter plots to distinguish risk patients among the risk features. The data above are from the TCGA cohort (tumor: *n* = 370). In addition, the validation set was obtained from the ICGC database (tumor: *n* = 240).

### 2.5. Mutational Analysis and Construction of a Nomogram Prediction Model for Risk Signature

The SangerBox 3.0 tool was used (https://doi.org/10.1002/imt2.36). Mutation differences in significant HCC genes between high- and low-risk groups were compared, and waterfall plots were plotted to present the results. The nomogram prediction model was based on multivariate Cox regression analysis and was used to predict 1-, 2-, and 3-year OS in patients with HCC. The calibration curve was used to predict survival in terms of the OS of HCC patients. Nomogram prediction model parameters included mRNA risk score, age, grade score, *T*, and stage. Time ROC curve and decision curve analysis (DCA) were employed to evaluate the accuracy of the nomogram prediction model and other parameters. The R software “timeROC,” “ggdca,” “survminer,” and “survival” packages were used for analysis and visualization.

### 2.6. Integrated Analysis of METTLs and mRNA Risk Signatures

The R software “pRRophetic” package was used and based on the drug IC50 values to screen sensitive drugs to METTLs and mRNA risk profiles [21]. Spearman correlation analysis of METTLs and mRNA risk signatures with EMT core genes, immune checkpoints, immune cells, tumor-associated macrophage (TAM) surface genes, TMB, MSI, RNAss, and IC50 were assessed using R software “reshape2” and “RColorBrewer” packages. Gene set variant analysis (GSVA) was employed to evaluate the biological functions of METTLs/mRNA risk signatures based on the gene sets of KEGG terms and hallmark terms.

### 2.7. Processing and Analysis of Single-Cell Data

The HCC single-cell dataset of this study (GSE146115, *n* = 4) was obtained from the GEO database. R software “Seurat,” “dplyr,” and “singleR” packages were used for quality control processing and subpopulation annotation of single-cell sequencing data in this study [22, 23]. Quality control conditions were set to retain genes expressed in at least three intracellular compartments, cells expressing at least 200 genes, cells with the number of detected genes between 200 and 2,500, and cells with less than 5% expression of mitochondrial genes. Lognormalize was used for normalization in this study. Cell communication was analyzed using the “sqjin/CellChat” package. Based on Genemania database construction (http://genemania.org/) [24] to construct a TME remodeling associated protein interaction network (PPI).

### 2.8. Statistical Analysis

R software 4.0.3 was used to perform all statistical analyses. Differences between the two groups were analyzed using the Wilcoxon rank sum test or paired *t*-test. Differences between METTLs and mRNA risk signatures and clinicopathological features were analyzed using the Wilcox test. *P* < 0.05 was considered statistically significant ( ^*∗*^*P* < 0.05,  ^*∗∗*^*P* < 0.01,  ^*∗∗∗*^*P* < 0.001,  ^*∗∗∗∗*^*P* < 0.0001).

## 3. Results

### 3.1. METTLs (METTL1/13/18/21A/23/25/2A/2B/5/6/9) are Upregulated in HCC and Have a Poor Prognosis for HCC Patients

**Figure 1:**
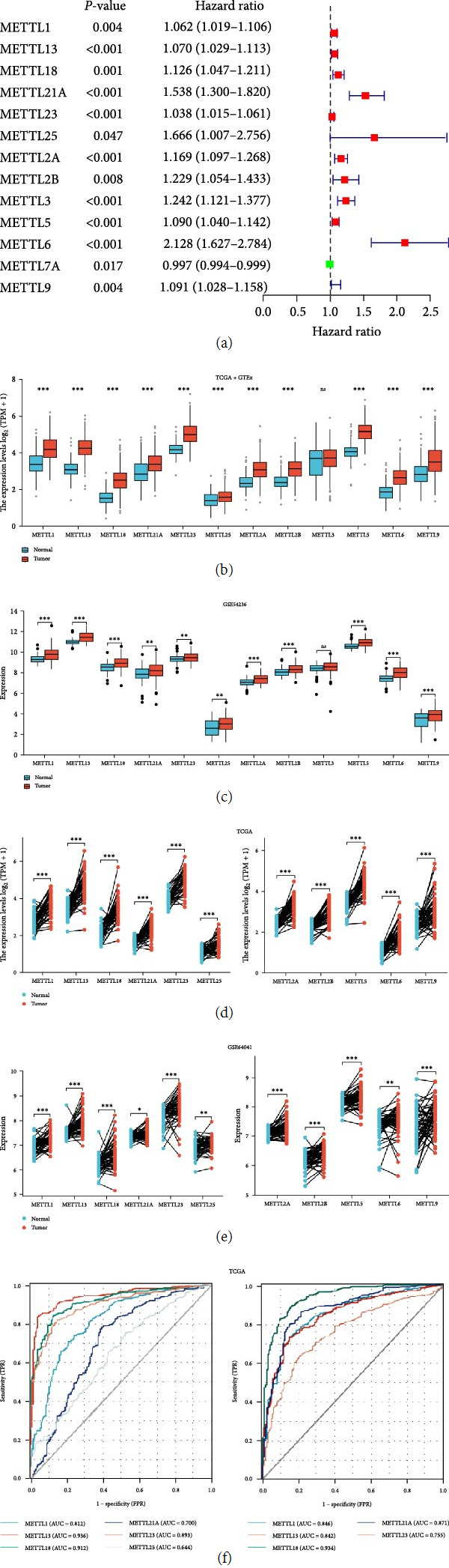
METTLs are upregulated in HCC and contribute to poor prognosis in HCC patients. (a) Univariate Cox analysis, (b) 12 mRNAs in TCGA + GTEX database were differentially expressed (tumor: *n* = 374, normal: *n* = 160), (c) the 12 mRNAs in the GSE54236 dataset were differentially expressed (tumor: *n* = 81, normal: *n* = 80), (d) pairwise difference analysis of 11 mRNAs in TCGA database (tumor: *n* = 50, normal: *n* = 50), (e) based on pairwise differential analysis of 11 mRNAs in the GSE64041 dataset (tumor: *n* = 60, normal: *n* = 60), and (f) ROC of METTLs in TCGA database.

To investigate the prognosis and expression of METTL family members in HCC. In this study, 34 METTL family members were first subjected to univariate Cox analysis, which revealed that 13 genes were prognostic for HCC patients (Figure 1(a)). Among them, 12 genes (METTL1/13/18/21A/23/25/2A/2B/3/5/6/9) showed a poor prognosis for HCC patients. This study subsequently combined TCGA with GTEx data to analyze the differential expression of these 12 genes in HCC. The results indicated that the expression of the remaining 11 genes (METTL1/13/18/21A/23/25/2A/2B/5/6/9), except METTL3, was significantly upregulated in HCC (*P* < 0.05) (Figure 1(b)). The same was true for GSE54236 results (Figure 1(c)). The paired analysis results of these 11 genes showed that cancer tissues had significantly higher expression than adjacent noncancerous tissues (Figures 1(d) and 1(e)). Moreover, the ROC curve results indicated that METTLs had significant diagnostic values (AUC > 0.6) for HCC patients (Figure 1(f)). In summary, METTLs (METTL1/13/18/21A/23/25/2A/2B/5/6/9) were enrolled as follow-up subjects in this study.

### 3.2. Expression Differences and Genetic Alterations of METTLs in Four Clinicopathological Features

**Figure 2:**
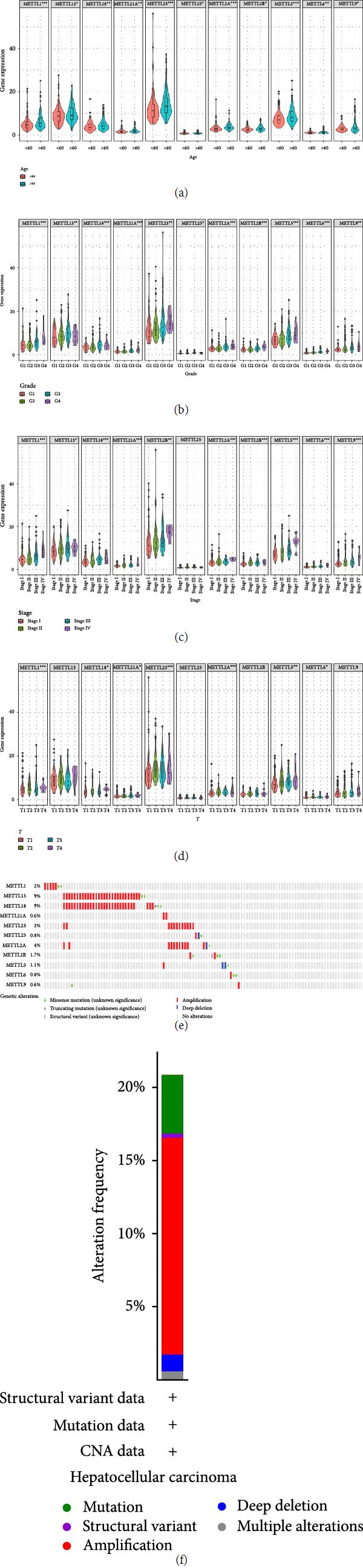
Expression differences and genetic alterations of METTLs in four clinicopathological features. (a–d) Differentially expressed METTLs with age, grade, stage, and *T*, (e) genetic alteration rate in METTLs, and (f) total genetic alteration rate in METTLs.

In this study, the expression of METTLs was differentially correlated with age, grade, stage, and *T*. The results showed that METTLs were significantly upregulated in patients older than 60 years (*P* < 0.05) (Figure 2(a)). For grade, the expression of METTLs was significantly different from grade (*P* < 0.05) (Figure 2(b)). For stage, except for METTL25, the expression of the other 10 genes was significantly different from stage (*P* < 0.05) (Figure 2(c)). For *T*, except for METTL13/25/2B/9, the expression of the remaining seven genes was significantly different from that of *T* (*P* < 0.05) (Figure 2(d)). The present study further evaluated the genetic alterations in METTLs. The results indicated genetic alterations in both METTLs (Figure 2(e)). In addition, 50% of the total genetic alterations were in these 11 genes (Figure 2(f)).

### 3.3. Prognostic Value of METTLs in HCC

**Figure 3:**
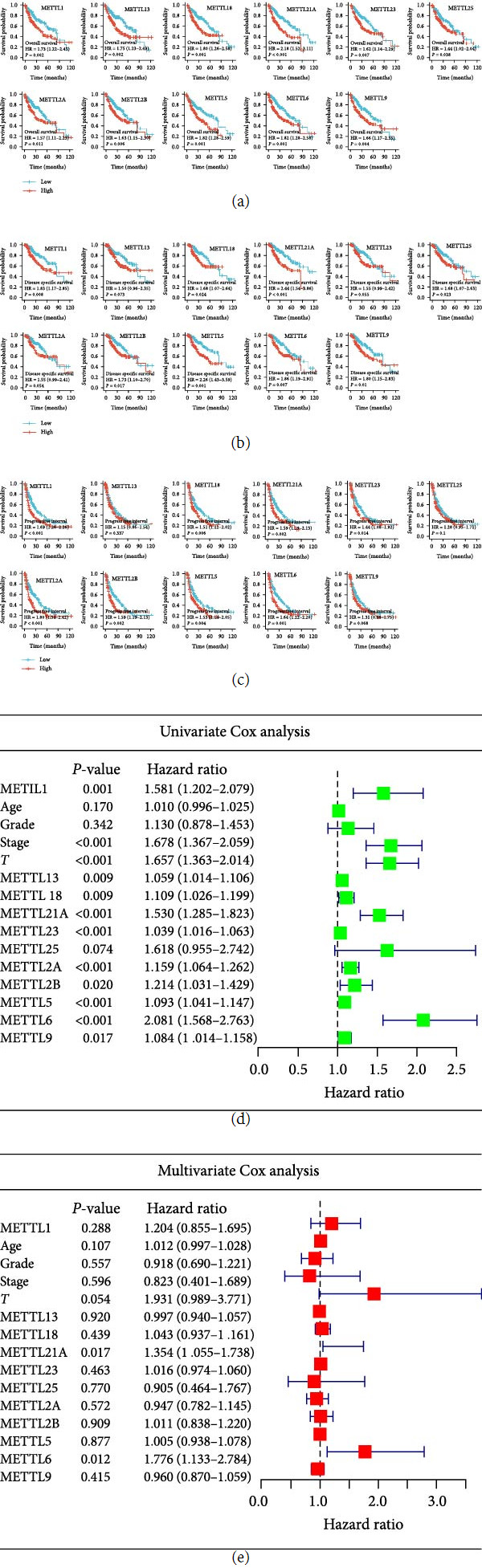
Prognostic value of METTLs in HCC. (a–c) Overall survival (OS), disease-specific survival (DSS), and progression-free survival (PFS) curves of METTLs; (d– and e) univariate/multivariate Cox analysis.

In this study, the prognostic value of METTLs in HCC was evaluated by OS, DSS, progression-free survival (PFS), and univariate/multivariate Cox analysis. OS results indicated that the high-expression group of METTLs had a significantly shorter survival time than the low-expression group (*P* < 0.05) (Figure 3(a)). The DSS results indicated that except for METTL13/23/2A, the high-expression group of the remaining eight genes had a significantly shorter survival time than the low-expression group (*P* < 0.05) (Figure 3(b)). The PFS results indicated that except for METTL13/25/9, the high-expression group of the remaining eight genes had a significantly shorter survival time than the low-expression group (*P* < 0.05) (Figure 3(c)). The results of univariate Cox analysis indicated that METTLs, *T* grade, grade, and stage were all prognostic factors for HCC patients (Figure 3(d)). We further included METTLs, age, *T*, grade, and stage in the multivariate Cox analysis. The results indicated that METTL21A/6 were independent prognostic markers for HCC patients (Figure 3(e)).

### 3.4. A Total of 664 mRNAs Showed a Strong Positive Correlation with METTLs

**Figure 4:**
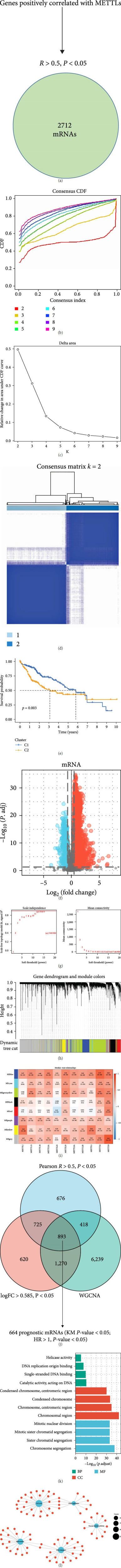
664 mRNAs are strongly and positively correlated with METTLs. (a) mRNAs with a significant positive correlation with METTLs, (b) cumulative distribution map of clustering consistency, (c) clustering Delta area map, (d) clustering results of the expression of METTLs on TCGA HCC samples, (e) OS curves of C1 and C2, (f) MRNA for difference between C1 and C2 (|logFC| > 0.585, *P* < 0.05), (g– and h) soft thresholding and dendrograms, (i) heatmap of correlation between module eigengenes and METTLs, (j) intersection genes and results of univariate Cox analysis, (k) GO analysis, and (l) KEGG analysis.

In this study, we screened mRNAs that were closely and positively correlated with METTLs. In this study, we first identified 2,712 mRNAs that were significantly and positively correlated with METTLs (Pearson *r* > 0.5 and *P* < 0.05) (Figure 4(a)). Subsequently. This study performed unsupervised consensus clustering of TCGA HCC samples according to the expression of METTLs. Based on CDF (Figure 4(b)) and delta area (Figure 4(c)), two clusters (C1 and C2) could be well separated when *k* = 2 (Figure 4(d)). OS results indicated that C1 had a higher survival time than C2 (Figure 4(e)). The present study screened differentially expressed mRNAs based on the two subpopulations C1 and C2 (|log FC| > 0.585, *P* < 0.05). In total, 417 downregulated and 3,508 upregulated mRNAs were screened out (Figure 4(f)). In this study, a coexpression network of METTLs was further constructed by WGCNA based on TCGA mRNA expression data. WGCNA soft threshold of 12 (Figure 4(g)). The present study identified eight mRNA coexpression modules (Figure 4(h)). We found that blue, cyan, yellow, purple, and gray modules were significantly associated with METTLs (Figure 4(i)). A total of 8,820 mRNAs were involved in these above modules. Ultimately, the related mRNAs of METTLs, the upregulated mRNAs in the subpopulation, and the coexpressed mRNAs in WGCNA were intersected. The intersection RNAs were subjected to univariate Cox analysis. We obtained a total of 664 mRNAs (Figure 4(j)). These mRNAs showed a close positive correlation with METTLs. GO and KEGG analyses were performed on these mRNAs. The biological functions of these RNAs were shown to be related to DNA replication, chromosome splicing, RNA transport, and cell cycle (Figures 4(k) and 4(l)).

### 3.5. Construction of an mRNA Risk Signature Associated with METTLs

**Figure 5:**
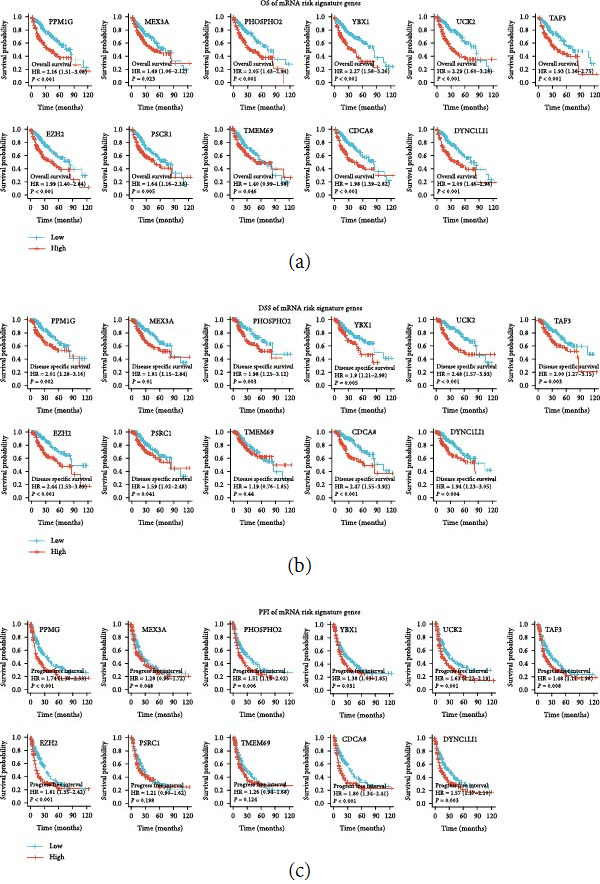
OS, DSS, and PFI curves of 11 mRNA risk signature genes. (a) OS curves, (b) DSS curves, and (c) PFI curves.

**Figure 6:**
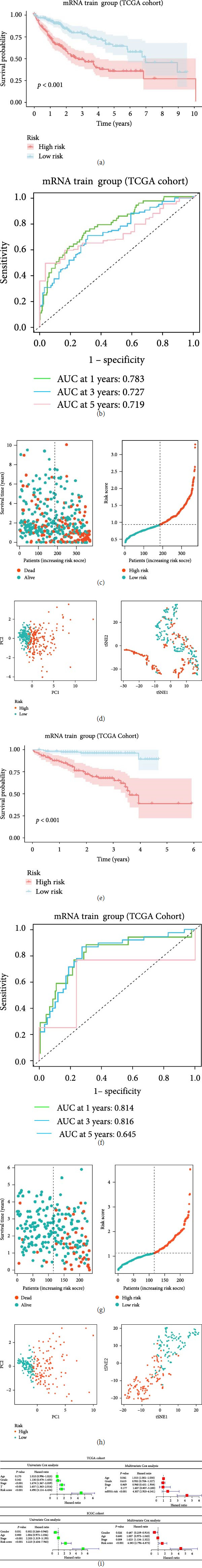
Analysis of mRNA risk signature. (a) Overall survival difference between high- and low-risk group in the mRNA training set, (b) time-dependent ROC curve in the mRNA training group, (c) high- and low-risk score median values and survival status distribution in the mRNA training set, (d) principal component analysis (PCA) and *t*-SNE scatter plots in the mRNA training set, (e) overall survival difference between high- and low-risk groups in the mRNA validation set, (f) time-dependent ROC curve in the mRNA validation set, (g) high- and low-risk score median values and survival status distribution in the mRNA validation set, (h) principal component analysis and *t*-SNE scatter plots in the mRNA validation set, and (i) univariate/multivariate Cox analysis of the training and validation sets.

In this study, based on 664 mRNAs associated with METTLs, the best mRNA risk signature was constructed by multivariate Cox regression analysis using lasso (Figures S1(a) and S1(b)). A total of 11 mRNAs (PPM1G, MEX3A, PHOSPHO2, YBX1, UCK2, TAF3, EZH2, PSRC1, TMEM69, CDCA8, and DYNC1LI1) were identified in this study to construct the best risk signature. To verify the reliability of these 11 mRNAs, the Heatmap of coexpression between the construct METTLs and these 11 mRNAs was performed using Spearman's correlation test. As shown in Figure S2(a), METTLs were significantly and positively correlated with these 11 mRNAs (*r* > 0.3, *P* < 0.05). Moreover, these 11 mRNAs were all upregulated in HCC (Figure S2(b)). Moreover, in the OS curve, the high expression of these 11 mRNAs showed a significant decrease in survival (*P* < 0.05) (Figure 5(a)). In the DSS curves, the survival of the high-expression group of mRNAs except TMEM69 decreased significantly (*P* < 0.05) (Figure 5(b)). In the PFI curves, the high expression of mRNAs except PSRC1 and TMEM69 showed a significant decrease in survival (*P* < 0.05) (Figure 5(c)). In conclusion, our resulting 11 mRNAs were reliable. Subsequently, we constructed mRNA risk signatures. The risk score was calculated as follows: Risk score (mRNA) = (0005  ^*∗*^ PPM1G exp) + (0007  ^*∗*^ MEX3A exp) + (0119  ^*∗*^ PHOSPHO2 exp) + (0001  ^*∗*^ YBX1 exp) + (0036  ^*∗*^ UCK2 exp) + (0068  ^*∗*^ TAF3 exp) + (0002  ^*∗*^ EZH2 exp) + (0031  ^*∗*^ PSRC1 exp) + (0009  ^*∗*^ TMEM69 exp) + (0003  ^*∗*^ CDCA8 exp) + (004  ^*∗*^ DYNC1LI1 exp). For the mRNA training set (TCGA) risk signature, the high-risk group showed significantly worse OS than the low-risk group (*P* < 0.05) (Figure 6(a)). The area under the curve (AUC) at 1, 3, and 5 years in the time-dependent ROC curve was 0.783, 0.727, and 0.719, respectively (Figure 6(b)). There were more deaths in the high-risk group (Figure 6(c)). PCA and *t*-SNE scatter plot results indicated that HCC patients at different risks were well able to be separated into two clusters (Figure 6(d)). The same was true for the mRNA validation set (ICGC) (Figure 6(e)–6(h)). Moreover, combined with clinicopathological features, univariate/multivariate Cox analysis indicated that the mRNA risk signature was an independent poor prognostic factor for HCC patients (Figure 6(i)).

### 3.6. Construction of an mRNA Risk Signature Nomogram Prediction Model

**Figure 7:**
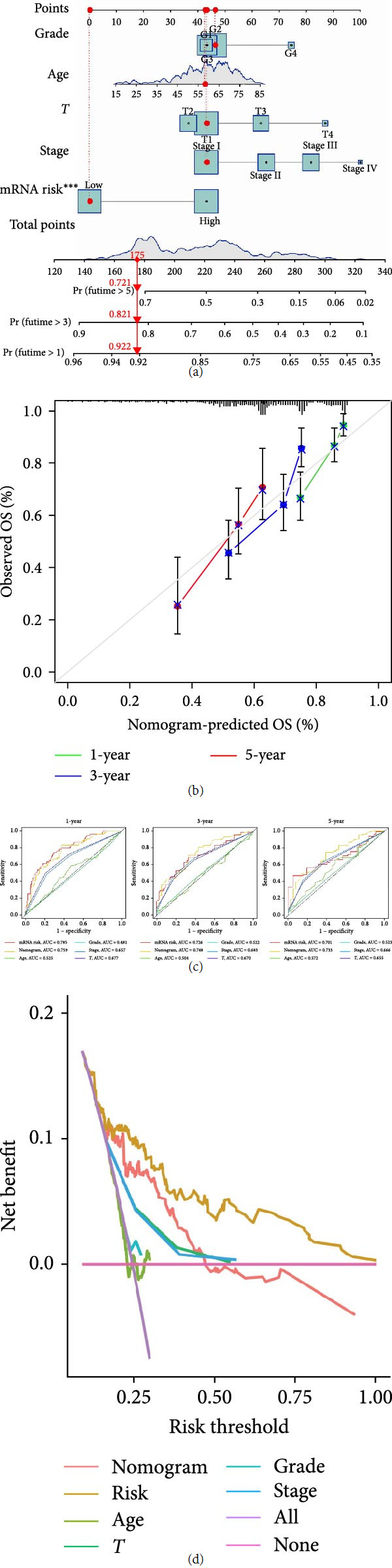
Construction of the mRNA risk signature nomogram prediction model. (a) Nomogram prediction model, (b) calibration curve, (c) the time ROC curves for the particular periods show the AUC values for the individual parameters, and (d) decision curve analysis (DCA).

In this study, a nomogram prediction model was constructed to evaluate the predictive value of the mRNA risk signature for the OS of HCC. In this study, a nomogram was constructed combining clinicopathological features and mRNA risk features to predict the survival rates of HCC patients with 1-, 3-, and 5-year OS (Figure 7(a)). The calibration curves indicated that the nomogram prediction model was able to predict the 1-, 3-, and 5-year OS of HCC patients with better accuracy (Figure 7(b)). The time ROC curves indicated that the 3- and 5-year nomogram prediction models outperformed the mRNA risk signature (Figure 7(c)). DCA indicated that the mRNA risk signature was more accurate (Figure 7(d)).

### 3.7. Correlation of mRNA Risk Signature with Clinicopathological Features and Genetic Alterations

**Figure 8:**
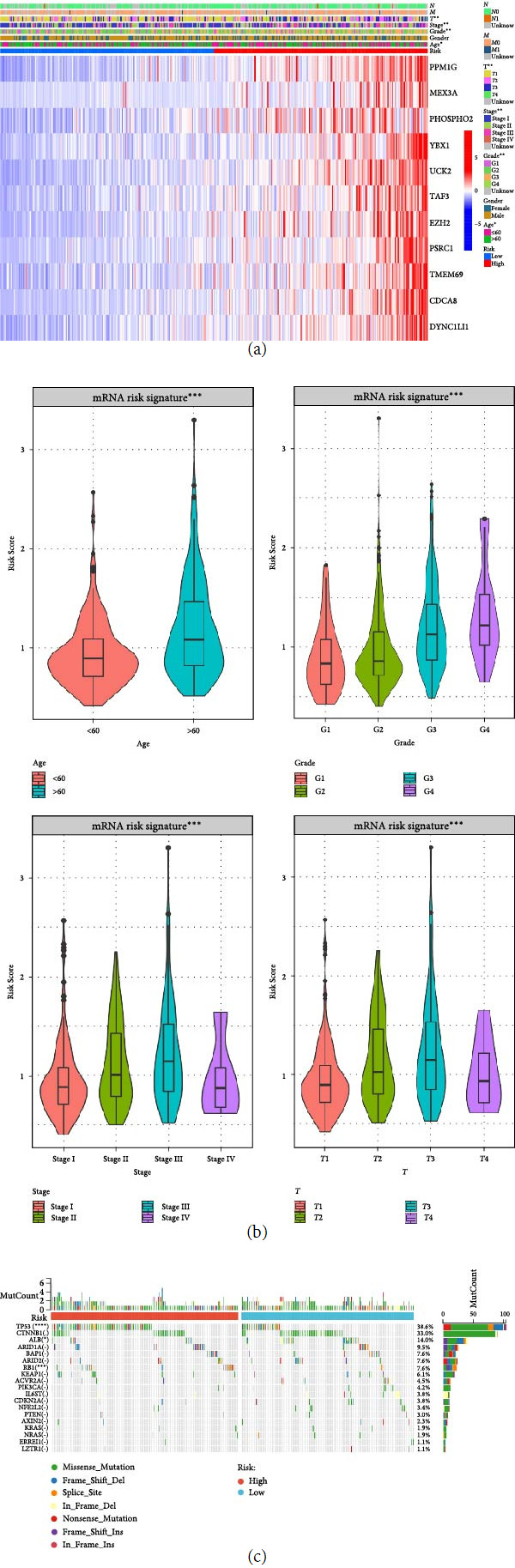
Correlation of mRNA risk signature with clinicopathological features and mutation difference analysis. (a) Differences between mRNA high- and low-risk groups and clinicopathological characteristics, (b) differences between mRNA risk scores and clinicopathological characteristics, and (c) mutational differences in mRNA high- and low-risk groups of the 20 mostly mutated genes in HCC.

This study evaluated the correlation of mRNA risk signature with clinicopathological features and genetic alterations. The results showed significant differences between *T*, grade, and stage with high- and low-risk groups of mRNAs (Figure 8(a)). In addition, mRNA scores were significantly different from age, grade, stage, and *t* (Figure 8(b)). In this study, we further compared the mutational differences between high- and low-risk groups in 20 genes (TP53, CTNNB1, ALB, AXIN2, KEAP1, BAP1, NFE2L2, LZTR1, RB1, PIK3CA, KRAS, IL6ST, CDKN2A, ARID2, ARID1A, ACVR2A, NRAS, HISR1H1C, PTEN, and ERRFI1) that are predominantly mutated in HCC [25]. The results showed that TP53 and RB1 mutations were significantly higher in the high-risk group (*P* < 0.05) (Figure 8(c)).

### 3.8. Multiangle Analysis of METTLs and mRNA Risk Signatures

**Figure 9:**
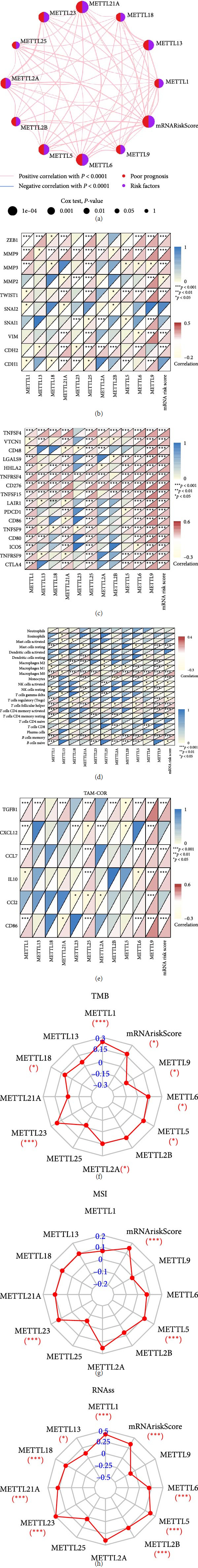
Multiangle analysis of METTLs and mRNA risk signature. (a) Risk network plot of therisk signature, (b) correlation of METTLs and mRNA risk scores with epithelial-mesenchymaltransition (EMT) core genes, (c) immune checkpoints, (d) immune cell infiltration, (e) tumor-associated macrophage cell markers, (f) tumor mutational burden (TMB), (g) microsatelliteinstability (MSI), and (h) tumor stem cells (RNAss), respectively.

This study further used multiple angles to assess the role of METTLs and mRNA risk signatures in HCC, including EMT, immune checkpoints, immune cell infiltration, TAM markers, TMB, MSI, and RNAss. The risk network plots of METTLs and mRNA risk signature were first constructed in this study (Figure 9(a)). The EMT results indicated that METTLs and mRNA risk scores were positively correlated with most of the EMT core genes, among which they were all significantly correlated with MMP9 (*P* < 0.05) (Figure 9(b)). The results of immune checkpoint analysis indicated that METTLs and mRNA risk score were significantly and positively correlated with most immune checkpoints (Figure 9(c)). Immune cell infiltration results indicated that METTLs and mRNA risk scores were positively correlated with multiple immune cell infiltration, among which all genes and mRNA risk scores except METTL25 were significantly correlated with M0 macrophages (*P* < 0.05) (Figure 9(d)). We further analyzed the correlations between the METTLs and mRNA risk scores and TAM markers. The results showed that the METTL9 and mRNA risk scores were significantly (*P* < 0.05) correlated with all TAM markers (Figure 9(e)). In addition, the METTL1/18/23/2A/2B/5/6/9 and mRNA risk scores were significantly positively correlated with TMB values (*P* < 0.05) (Figure 9(f)). There was a significant positive correlation between METTL23/5 and mRNA risk score and MSI (*P* < 0.05) (Figure 9(g)). The METTL1/13/18/21A/23/2A/2B/5/6 and mRNA risk scores showed significant positive correlations with RNAss (*P* < 0.05) (Figure 9(h)). In conclusion, METTLs and mRNA risk signature play a crucial role in HCC and may serve as novel therapeutic targets in tumor therapy.

### 3.9. Assessment of Potential Pathways for the METTLs and mRNA Risk Signature

**Figure 10:**
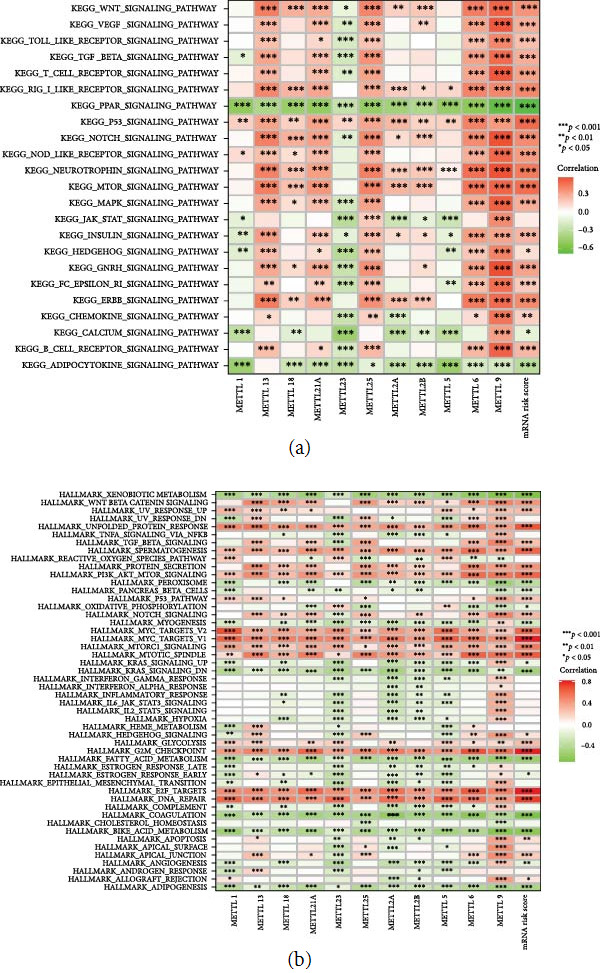
Correlation analysis of METTLs and mRNA risk signature with potential pathways. (a) KEGG terms calculated by GSVA and (b) GSVA calculated hallmark terms.

The present study further evaluated the potential pathways of METTLs and mRNA risk score in HCC. Based on KEGG terms calculated by GSVA, we found that METTLs and mRNA risk scores were significantly associated with multiple tumors- or immune-related pathways (*P* < 0.05) (Figure 10(a)). As the Wnt pathway was significantly positively correlated with mttl18/21A/25/2A/2B/6/6 and mRNA risk score; there were significant positive correlations between VEGF pathway and METTL21A/25/2B/6/9 and mRNA risk score; P53 pathway was significantly and positively correlated with METTL1/13/18/21A/23/25/2A/2B/5/6/9 and mRNA risk score. In addition, the hallmark terms, METTLs, and mRNA risk scores calculated based on GSVA were significantly associated with multiple tumors- or immune-related terms (*P* < 0.05) (Figure 10(b)). These terms include the terms: “XENOBIOTIC_METABOLISM,” “WNT_BETA_CATENIN_SIGNALING,” “UNFOLDED_PROTEIN_RESPONSE,” “TNFA_SIGNALING_VIA_NFKB,” “TGF_BETA_SIGNALING,” “SPERMATOGENESIS,” “PROTEIN_SECRETION,” “PI3K_AKT_MTOR_SIGNALING,” “MYC_TARGETS_V1,” “MYC_TARGETS_V2,” “K_MTORC1_SIGNALING,” “MITOTIC_SPINDLE,” and so forth.

### 3.10. Screening for Sensitive Drugs Shared with METTLs and mRNA Risk Signatures

**Figure 11:**
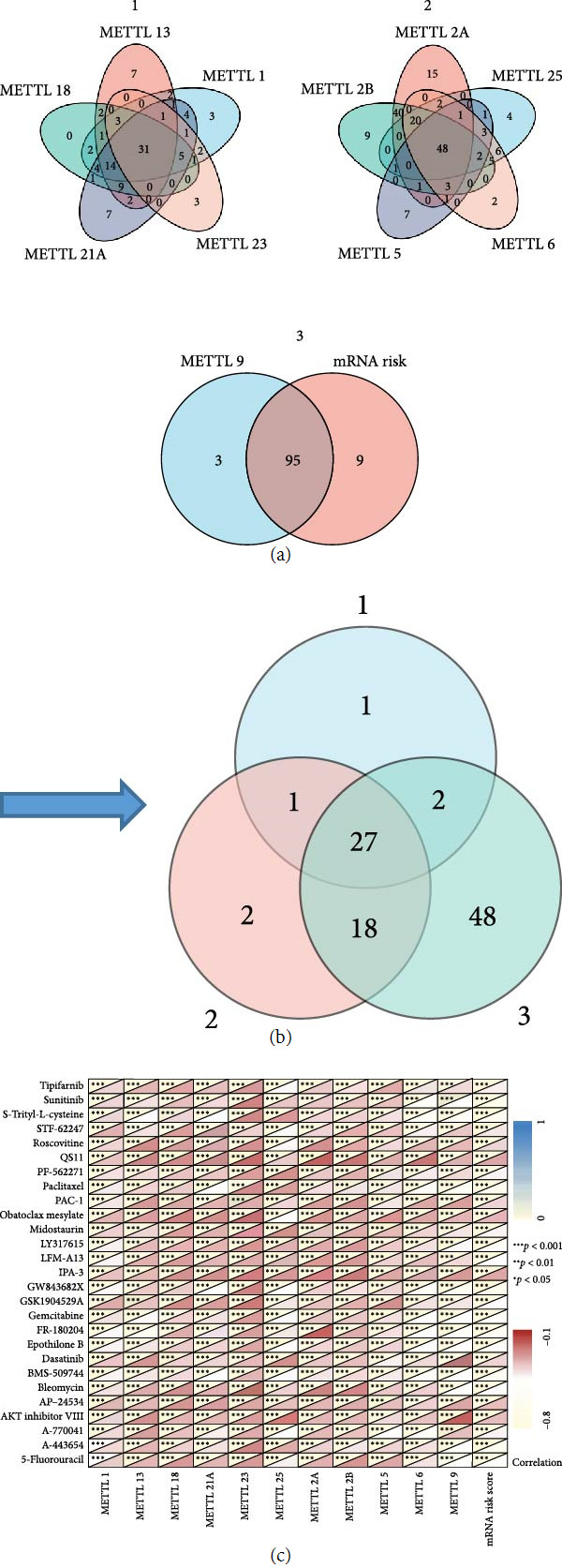
Screening for sensitive drugs shared with METTLs and mRNA risk signatures. (a and b) Screening for drugs in common with METTLs and mRNA risk profiles; (c) Heat map of correlation between IC50 values of 27 drugs and METTLs and mRNA risk scores.

This study evaluated the correlation of METTLs and mRNA risk profiles with drug IC50 and screened for common-acting sensitive drugs. We selected drugs with significant associations, including 31 drugs in common with METTL1/13/18/21A/23, 48 drugs in common with METTL25/2A/2B/5/6, and 95 drugs in common with METTL9 and the mRNA risk signature (Figure 11(a)). All three had 27 drugs in common with significant associations with METTLs and mRNA risk scores (Figure 11(b)). Moreover, the IC50 values of these 27 drugs were all significantly inversely correlated with the METTLs and mRNA risk scores (*P* < 0.05) (Figure 11(c)). Therefore, these 27 drugs had higher sensitivity for METTLs and mRNA risk signature.

### 3.11. Quality Control of Single-Cell Sequencing Data Relates to Cell Subpopulation and Constructs a PPI Network Related to tumor Microenvironment Remodeling

**Figure 12:**
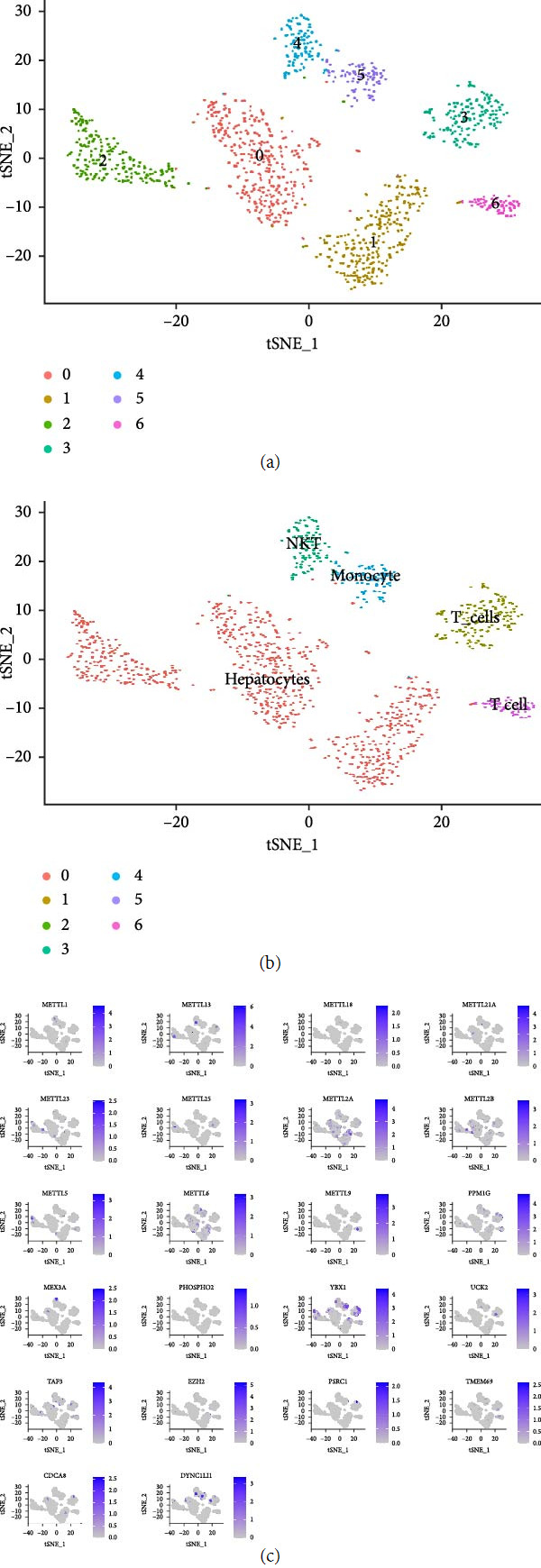
Cell subpopulations for single-cell sequencing data. (a) Seven with differential cell populations, (b) after annotating the four cell populations (*T* cells, NK cells, monocytes, and liver parenchymal cells), and (c) expression distribution of METTL1/13/8/21A/23/25/2A/2B/5/6/9 and 11 mRNA risk signature genes in these four types of cells.

**Figure 13:**
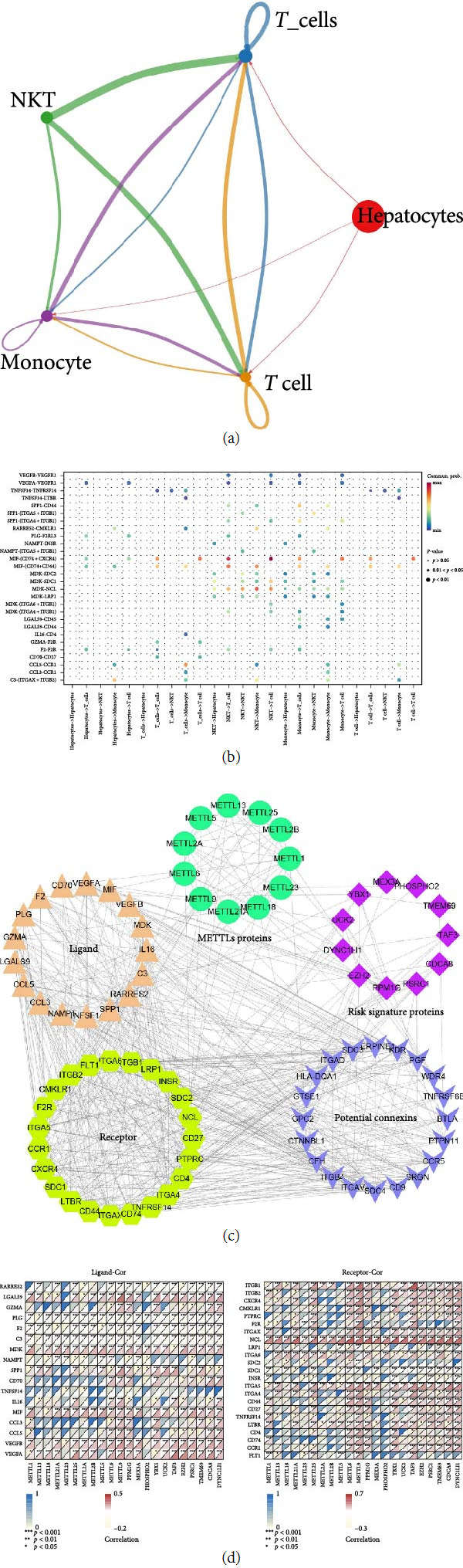
Construction of a PPI network related to tumor microenvironment (TME) remodeling. (a and b) Crosstalk between *T* cells, NK cells, monocytes, and liver parenchymal cells linking and corresponding ligands, (c) PPI network, including 11 METTLs proteins (circles), 11 mRNA risk signature proteins (diamonds), receptors (hexagons), ligands (triangles), and potential connexins (*V*-shapes), and (d) correlation of ligands to METTL1/13/18/21a/23/25.

The HCC single-cell sequencing dataset gse146115 (*n* = 4) was first quality-controlled and filtered in this study. Gene expression profiles of 1,263 high-quality cells were obtained for this study (Figure S3(a)). In addition, we obtained 2,000 variable genes for cell binning and annotation (Figure S3(b)). Subsequently, following PCA dimensionality reduction (Figure S3(c)) and differential analysis in this study, we obtained seven cell populations (Figure 12(a)). Finally, after “singleR” package annotation, we derived four cell populations (*T* cells, NK cells, monocytes, and liver parenchymal cells) (Figure 12(b)). METTL1/13/18/21A/23/25/2A/2B/5/6/9 and 11 mRNA risk signature genes were present to varying degrees in these four types of cells (Figure 12(c)). Because of the crosstalk connections among these four cell populations (Figure 13(a)), we predicted the receptors and ligands between them (Figure 13(b)). Subsequently, we established a PPI network including METTL1/13/18/21A/23/25/2A/2B/5/6/9 (circles), 11 mRNA risk signature proteins (diamonds), receptors (hexagons), ligands (triangles), and potential connexins (*V*-shapes) (Figure 13(c)). This PPI network is associated with the communication between these four types of cells (*T* cells, NK cells, monocytes, and liver parenchymal cells). In addition, METTL1/13/18/21A/23/25/2A/2B/5/6/9 and mRNA risk signature genes showed significant correlations with receptors and ligands for these four cells (Figure 13(d)). Therefore, this PPI network is associated with TME remodeling.

## 4. Discussion

HCC is a malignant tumor, and the pathogenesis remains unclear to date [26]. HCC has a high mortality rate, mainly because most patients are advanced when diagnosed or have developed local metastases [27]. There are many therapeutic approaches for HCC, including early diagnosis, surgery, drugs, immunotherapy, and so forth. However, current treatments do not achieve the desired efficacy for patients with advanced HCC stages [28]. Therefore, it is crucial to find out effective biomarkers and therapeutic targets for HCC. In the present study, METTLs (METTL1/13/18/21A/23/25/2A/2B/5/6/9), among 33 METTL family members are poor prognostic factors in HCC patients and upregulated in. In addition, METTLs have a better ability to discriminate between tumor and nontumor. Therefore, we focused on these 11 genes as research objects and comprehensively analyzed their values for HCC.

In the present study, OS results indicated that the METTLs high-expression group had shorter; the DSS results indicated a shorter survival time in the high-expression group of the remaining eight genes, except for METTL13/23/2A; the PFS results indicated a shorter survival time in the high-expression group of the remaining eight genes except for METTL13/25/9. Univariate Cox analysis indicated that both METTLs were poor prognostic factors in HCC. Combined with multiple HCC clinicopathological features, multivariate Cox analysis indicated that METTL21A/6 was an independent prognostic marker for HCC. Therefore, overall METTLs are poor prognostic for HCC. Previous studies have shown that the probability of having HCC increases progressively with age as well [29]. Changes in the stage or grade of HCC can also be accompanied by alterations in specific regulated genes [30]. Invasion depth (*T*) is also one of the significant factors contributing to the poor prognosis of HCC patients [31]. In the clinical relevance analysis of this study, METTLs were significantly upregulated in patients older than 60 years. For the HCC grade, the expression of METTLs was significantly different from the grade, and the higher grade increased the expression of METTLs. For stage, except for METTL25, the expression of the remaining 10 genes increased with stage. For *T*, except for METTL13/25/2B/9, the expression of the remaining seven genes was significantly different from that of *T*. Moreover, 50% of the total genetic alterations in METTLs have been reported. Currently, METTL21A/23/25/2A/2B/9 are not studied in HCC. However, in other tumors, METTL2a is a potential N3 methylcytidine (M3C)—the related gene that promotes breast cancer development [32]. The rise of METTL9 is associated with peritoneal metastasis of gastric cancer [33]. In multiple previous studies, METTL1/13/18/5/6 were able to promote HCC progression as oncogenes. Chen et al. [10] showed that the knockdown of METTL1 can reduce m7G tRNA modification, reducing the translation of mRNA and thereby inhibiting HCC development. Decreasing METTL1 also reduces HCC recurrence [34]. Li et al. [35] showed that upregulation of METTL13 could promote HCC growth and metastasis. Downregulation of METTL18 can inhibit the proliferation and metastasis of HCC [17]. Peng et al. [36] showed that METTL5 is associated with 18S rRNA m6A modification, and downregulating METTL5 can inhibit HCC progression. Bolatkan et al. [12] showed that downregulating METTL6 could reduce metastasis of HCC. Taken together, METTLs have an essential role in HCC prognosis and progression.

In this study, 11 mRNA profiles of METTLs-related risk features were constructed by multiple algorithms. All these 11 mRNAs were closely associated with METTLs. These 11 mRNAs were upregulated in HCC and were poor prognostic factors for HCC. The mRNA risk signature could well stratify HCC patients into high- and low-risk groups. In the present study, we found that the mRNA risk signature was an independent poor prognostic factor for HCC patients. In clinical correlation analysis, the mRNA risk signature was associated with age, grade, stage, and *T* in HCC patients. In addition, the nomogram prediction model we constructed had high accuracy for OS 1, 3, and 5 years in patients with HCC. This nomogram prediction model had higher 3- and 5-year accuracies than the mRNA risk signature. However, DCA indicated more robust accuracy of mRNA risk signature. In the mutation analysis, we found that TP53 and RB1 were mutated with increased frequency in the high-risk group. It is well known that TP53 and RB1 mutations are one of the most common inheritances in HCC, and the more TP53 mutations, the worse the prognosis of HCC patients [37, 38]. Therefore, the mRNA risk signature we constructed has an essential effect on the prognosis and development of HCC. Previous studies have shown that seven mRNAs in the mRNA risk signature can act as oncogenes to promote HCC progression, including PPM1G [39], PHOSPHO2 [40], YBX1 [41], PSRC1 [42], UCK2 [43], EZH2 [44], and CDCA8 [45]. However, MEX3A, TAF3, TMEM69, and DYNC1LI1 have not been experimentally studied in HCC. However, in other tumors, they are also able to act as oncogenes to promote tumor progression [46–49]. These studies further illustrate that the mRNA risk signature we constructed is an important factor that promotes HCC occurrence. Further integrative analysis in this study found that METTLs and mRNA risk signatures were associated with EMT, multiple immune checkpoints, multiple immune cell infiltration, TMB, MSI, RNAs, and cancer pathways. In terms of drug sensitivity, 27 drugs sensitive to METTLs and mRNA risk profiles were screened in this study. In conclusion, this study provides a theoretical basis for HCC prognosis, progression, and drug screening.

Tumor production causes alterations or accumulation of surrounding immune or nonimmune cells [50]. These immune and nonimmune cells, some noncellular components, and the environment that tumor cells share is called the TME [51]. The TME has a dual role in that it can both inhibit tumor cell growth and allow tumor cells to evade surveillance and then promote tumor cell metastasis [52]. The TME also influences HCC progression and prognosis [53]. Previous studies have demonstrated that METTLs can influence the TME and immune therapy. For example, Liu et al. [54] showed that METTL1 is a key immune regulator in the TME, and targeting METTL1 can inhibit MDSC recruitment and enhance anti-PD-1 efficacy. Xu et al. [55] found that knocking down METTL5 can downregulate the expression of PD-L1, thereby inhibiting immune evasion in HCC. Zheng et al. [56] demonstrated that a model constructed with METTL9 can predict the immune therapy and prognosis of HCC. However, the impact of the remaining eight METTLs on the TME and immune therapy requires further investigation. In the present study, four cell populations were identified by single-cell data, including liver parenchymal cells, monocytes, NK cells, and *T* cells. In the present study, based on the communication between these four types of cells, we predicted the receptors and ligands between these four types of cells. Previous studies show that *T* cells and NK cells show that ligand imbalance leads to immune escape against tumor cells [57, 58]. Furthermore, targeting monocytes can enhance the therapeutic effect of HCC [59]. Therefore, a PPI network including METTLs, 11 mRNA risk signature proteins, four cellular receptors and ligands, and potential connexins was established in this study. This PPI network was associated with the communication between these four types of cells. In addition, the METTLs and mRNA risk signature genes were significantly associated with the receptors and ligands of these four cells. Therefore, this PPI network is associated with TME remodeling.

In summary, this study performed a multiangle comprehensive analysis of METTLs (METTL1/13/18/21A/23/25/2A/2B/5/6/9) and their mRNA risk signature, and these results provide a theoretical basis for HCC prognosis, biomarker screening, mechanism, and drug screening.

## Data Availability

Publicly available datasets were analyzed in this study. These can be found in the GEO database (https://www.ncbi.nlm.nih.gov/geo), Genotype-Tissue Expression (GTEx) (https://www.gtexportal.org/home/index.html), International Cancer Genome Consortium (ICGC) (https://dcc.icgc.org/), and The Cancer Genome Atlas (TCGA) (https://portal.gdc.cancer.gov). The original contributions presented in the study are included in the article/Supplementary Material. Further inquiries can be directed to the corresponding author.

## Supplementary Materials

Supplementary Materials Figure S1: the least absolute shrinkage and selection operator (lasso). Figure S2: coexpression analysis of METTLs with 11 mRNA risk signature genes and differential expression analysis of 11 mRNA risk signature genes. Figure S3: quality control of single-cell sequencing data.
