# Supplementary material for: Comprehensive Analysis of METTLs (METTL1/13/18/21A/23/25/2A/2B/5/6/9) and Associated mRNA Risk Signature in Hepatocellular Carcinoma
Source: Anal Cell Pathol (Amst). 2023 Oct 12;2023:6007431. doi: 10.1155/2023/6007431 (PMC10735724; doi:10.1155/2023/6007431)
Supplement: Supplementary Materials — Figure S1: the least absolute shrinkage and selection operator (lasso). Figure S2: coexpression analysis of METTLs with 11 mRNA risk signature genes and differential expression analysis of 11 mRNA risk signature genes. Figure S3: quality control of single-cell sequencing data. [file 6007431.f1.docx]

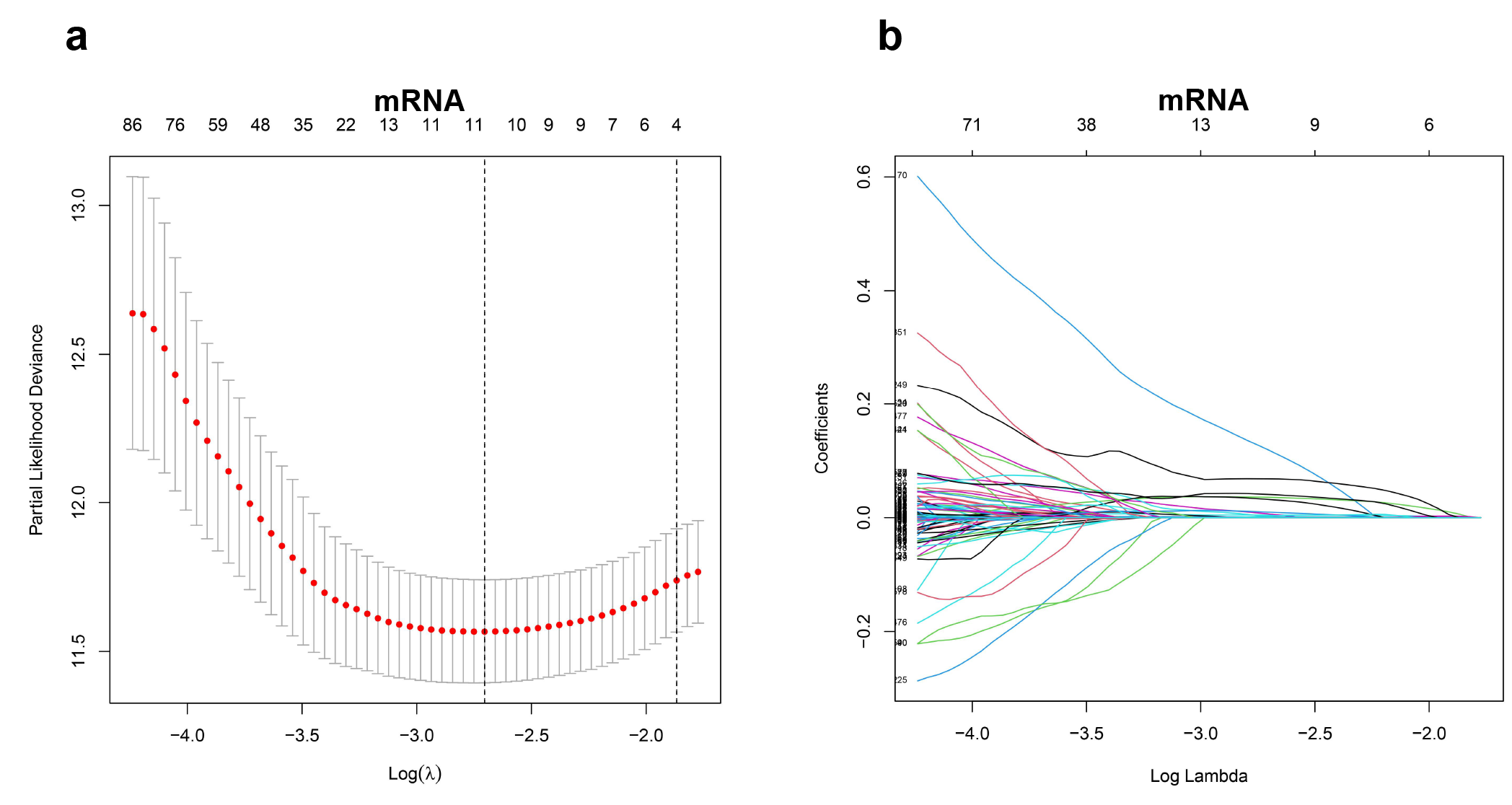


**Figure S1:** The least absolute shrinkage and selection operator (lasso). (a) Ten-fold cross-validations for screening of the optimal parameter (lambda). (b) LASSO coefficient profiles determined by the optimal lambda.
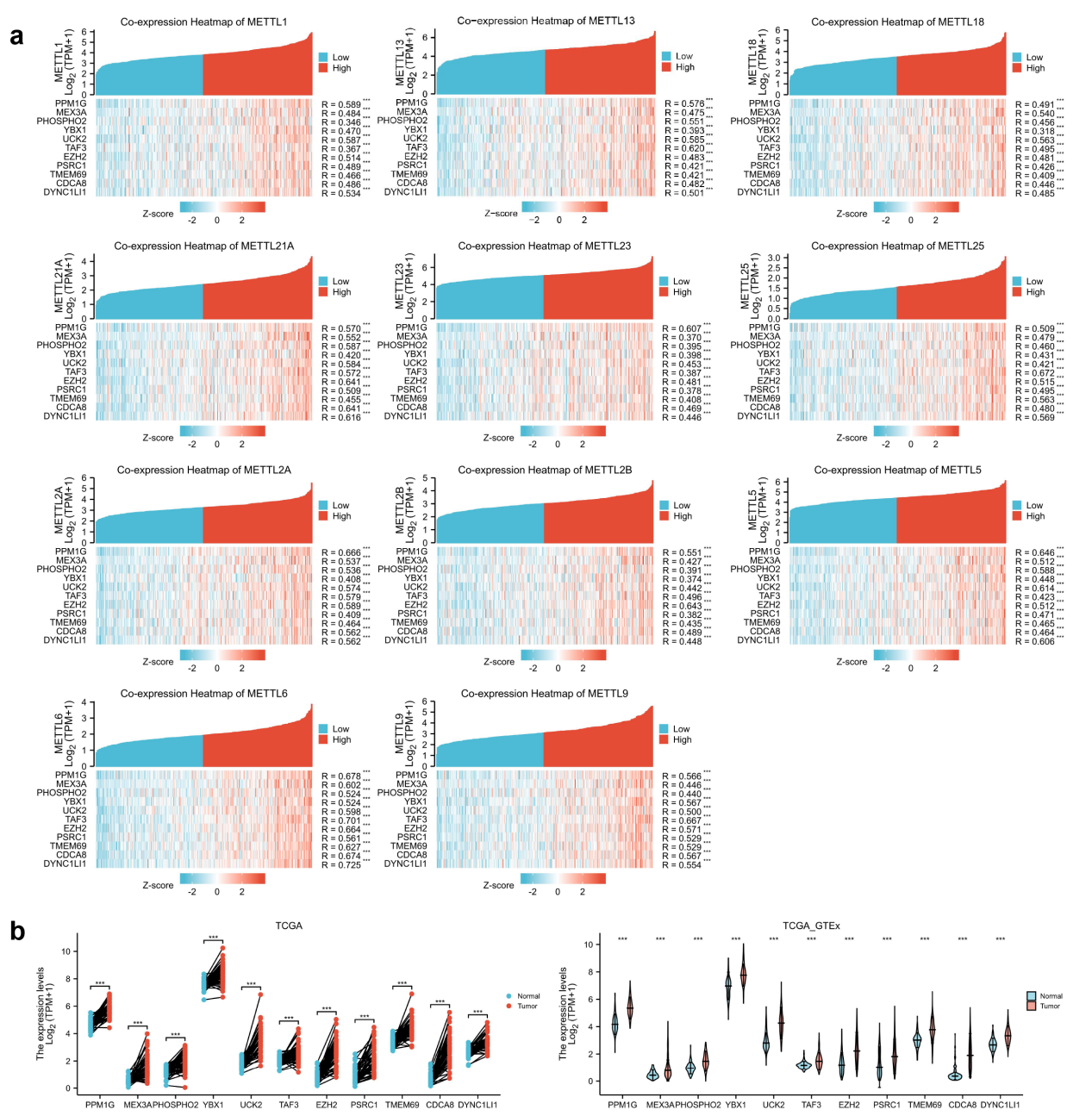


**Figure S2:** Co-expression analysis of METTLs with 11 mRNA risk signature genes and differential expression analysis of 11 mRNA risk signature genes. (a) Co-expression analysis of METTLs with 11 mRNA risk signature genes. (b) Differential expression analysis of 11 mRNA risk signature genes.
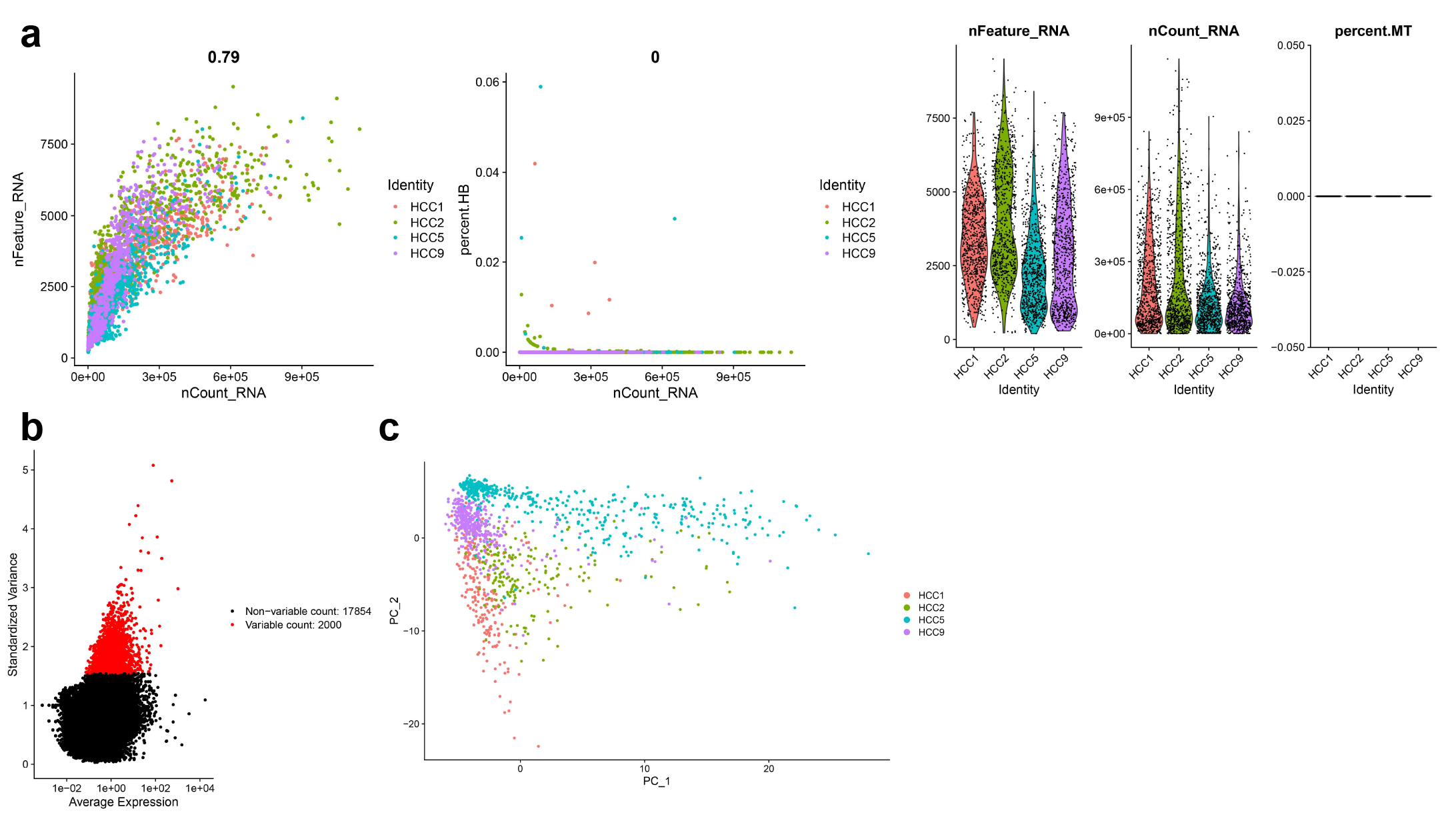


**Figure S3:** Quality control of single-cell sequencing data. (a) Gene expression profiles of 1263 high-quality cells were obtained for this study. (b) 2000 variable genes. (c) Principal component analysis (PCA).
